# First Lines of Education

**Published:** 1840-07

**Authors:** 


ART. II.—*First Lines of Education: a Course of Four Lectures delivered to the Members of the Literary and Scientific Institution, Worcester.* By EDWARD ASTBURY TURLEY, Surgeon.—*Worcester,* 1839. 8vo, pp. 81.

THE great increase of popular works, and the frequent delivery of lectures in every large town, aiming at the inculcation of sound principles of education, prove most satisfactorily that the period is not far distant when the empirical and fanciful pedagogue will be displaced by the rational and scientific instructor.

For some years enlightened teachers have considered it necessary to give their pupils due insight into the general laws governing their own organic structure, and the period is not far distant when another advance will be made, and the grand doctrine will be universally taught, that man's moral, intellectual, and animal faculties are solely dependent upon a portion of his organic structure. When the plain and simple truths of physiology are made to sweep away the present system, the result of metaphysical speculation; when the teacher is enabled to apply certain general and immutable laws in his course of education, instead of depending upon opinions and dogmas resulting from imperfect views of human nature; when, in fact, philosophy is advanced to the post hitherto occupied by empiricism—then, and not till then, will our youth be educated in accordance with and not in opposition to nature's commands.

Mr. Turley is a phrenologist, and his lectures were delivered for the purpose of "demonstrating how the physiology of the brain, or *phrenology* as it has been called, is applicable to the successful training of body and mind, called education." The first three lectures contain considerable information in reference to physical education. The fourth is very unsatisfactory as regards the application of phrenology, and Mr. T. by no means treats the subject of mental education in the philosophical manner we were led to expect.

We extract the following table, giving the results of eleven measurements of the infant head, and think a continuation of the investigation will afford some curious and valuable facts. The measurements were taken weekly, commencing upon the day of birth.

| | Nov. 23 | Nov. 30 | December 7 | December 14 | December 21 | December 28 | January 4 | January 11 | January 18 | Feb. 25 | March 4 |
|---|---|---|---|---|---|---|---|---|---|---|---|
| | In. | In. | In. | In. | In. | In. | In. | In. | In. | In. | In. |
| The circumference of the head | 13 | 14 | 14 | 14¼ | 14¼ | 14½ | 14½ | 14¾ | 15 | 15 | 15 |
| Space between the orifices of the ears | 7¾ | 8 | 8¼ | 8¾ | 8¾ | 9¼ | 9½ | 9¾ | 9¾ | 9¾ | 10 |

Speaking of this phrenologically Mr. T. says,—

"The increase in the respective regions, namely, the perceptive, reflective, moral, and animal were in this order: first the perceptive were seen to come forth, then the reflective, then the animal, then the moral. After this, and during the next six months, we noticed the onward movement to be in this order: the reflective, the animal, the moral, and then the perceptive; and with the jetting forth of the ears by destructiveness and combativeness, we had evidence enough of what we had never seen before, namely, passion or temper, as it is called, at opposition, even to striking of the offending object. (p. 48.)

Mr. T. believes the fact of the external division of the olfactory nerves terminating in the anterior extremity of the middle lobe of the cerebrum, to be a discovery of his own. This is not the case. Phrenologists have long noticed this distribution, and it has been described by anatomists for a very long period. We have no doubt these lectures, although not of a high order, were beneficial; and we are glad to see the members of our profession exerting themselves to remove the ignorance which surrounds the all-important subject of education.

